# Misinformation in YouTube Videos About Chiropractic Treatment for Otitis Media

**DOI:** 10.7759/cureus.93728

**Published:** 2025-10-02

**Authors:** Luke Reardon, Erin M Gawel, Shiven Sharma, Deepthi S Akella, David Riccio, Michele M Carr

**Affiliations:** 1 Otolaryngology - Head and Neck Surgery, Lincoln Memorial University - DeBusk College of Osteopathic Medicine, Knoxville, USA; 2 Otolaryngology, Jacobs School of Medicine and Biomedical Sciences, University at Buffalo, Buffalo, USA; 3 Otolaryngology, Icahn School of Medicine at Mount Sinai, New York, USA; 4 Otolaryngology, University of Missouri, Columbia, USA; 5 Otolaryngology, University of Connecticut, Hartford, USA

**Keywords:** chiropractic treatment, medical misinformation, otitis media, social media, youtube

## Abstract

Introduction: This study analyzes the prevalence and characteristics of misinformation in YouTube videos about chiropractic treatment for otitis media (OM).

Methods: YouTube was searched in January 2023 (Incognito mode, US region) using the terms “chiropractic treatment for otitis media”, “chiropractic ear infection”, and “chiropractic ear problems”. The first 50 English-language videos ranked by relevance were evaluated. Two independent reviewers extracted metadata (views, duration, likes, comments, upload source) and coded for references to evidence-based therapies, chiropractic techniques, and misinformation themes (“fixing” nerves or the Eustachian tube); a third reviewer resolved discrepancies. Descriptive statistics summarized video characteristics and engagement. A parallel PubMed search identified published literature on the most commonly mentioned techniques.

Results: Fifty videos accrued 2,600,209 views, with a mean of 192 seconds, and generated 21,102 likes and 1,766 comments. Chiropractors produced 42 videos (84% of the content); hospital or academic channels contributed two videos (4%). Only three videos (6%) cited scientific sources. Twenty-five videos claimed that chiropractic manipulation could “fix” the Eustachian tube, and 14 videos (28%) asserted nerve correction; none mentioned antibiotics or tympanostomy tubes. Upper‑cervical adjustments (32 videos, 64%) and ear‑massage maneuvers (25 videos, 50%) were the most frequently promoted techniques, despite limited or low‑quality supporting evidence in published literature.

Conclusions: Misinformation about chiropractic treatment for OM is widespread and highly viewed on YouTube. The omission of proven therapies and promotion of unverified claims pose risks for delayed care and preventable harm. Efforts from clinicians, professional societies, educators, and platforms are needed to elevate accurate content, promote media literacy, and reduce exposure to misleading medical information.

## Introduction

The proliferation of social media has transformed how people access and consume health information, with YouTube emerging as a particularly influential platform. While this democratization can empower individuals, it also poses significant risks when the disseminated content is inaccurate or misleading. This is especially concerning for medical treatments, where misinformation can have serious public health implications. Otitis media (OM) is a common middle ear infection and the second most common pediatric diagnosis in the emergency room following upper respiratory infections [1]. These infections are often treated with antibiotics, typically amoxicillin, or with surgical interventions in cases of persistent, recurrent, or complicated infections [2]. However, alternative treatments, such as chiropractic care, have gained attention. Proponents claim that spinal manipulations can alleviate or cure OM, but these assertions lack robust scientific validation [3-5] and are often spread via platforms such as YouTube [6], potentially misleading individuals seeking relief.

Social media platforms are a double-edged sword in the realm of health information dissemination [6]. While they offer unparalleled access to information, the lack of regulation can lead to the spread of unverified and potentially harmful medical advice [7,8]. The intersection of chiropractic treatment claims for OM and the influential power of YouTube as an information source necessitates a critical examination. Previous studies have shown that health-related videos on YouTube vary greatly in quality, with many presenting non-evidence-based information [9].

The objective of this cross-sectional descriptive study was to quantify misinformation in YouTube videos about chiropractic management of OM and assess its potential implications for caregiver decision-making and public health. This work addresses a notable gap in the literature regarding the accuracy of OM-related information on social media. Empirical data on misinformation can guide clinicians, policymakers, and platform administrators in promoting reliable content and limiting unverified medical claims. A clearer understanding of the scope and nature of misinformation will support the development of targeted education strategies and regulatory interventions that protect vulnerable pediatric populations and their caregivers in an increasingly digital world.

## Materials and methods

This research was reviewed by the University at Buffalo Institutional Review Board and determined to be non-human-subjects research (STUDY00005687). The study used a cross-sectional descriptive analysis to assess the prevalence and characteristics of misinformation in YouTube videos related to OM [10].

In January 2023, YouTube was searched using Google Chrome (United States region, cookies cleared, Incognito mode) with the keywords: “chiropractic treatment for otitis media”, “chiropractic ear infection”, and “chiropractic ear problems”. Incognito mode was used to limit bias from personalized search results. The first 50 English-language videos related to chiropractic treatment for OM, ranked by relevance by YouTube’s algorithm, were included in this study.

The sample was limited to the first 50 relevance-ranked, English-language videos to reflect the content most likely encountered by caregivers in a typical YouTube search. While this approach improves ecological validity by mirroring real-world user behavior, it may not capture the full spectrum of chiropractic-related videos on OM. Furthermore, YouTube’s search algorithm and video availability evolve over time, meaning that exact replication of these results at a later date may not be possible.

Data extracted from each video included the URL, title, duration in seconds, total number of views, upload date, number of likes, number of comments, whether the video referenced scientific sources, mentions of established treatments such as antibiotics or ear tubes, specific chiropractic techniques mentioned, claims about fixing nerves or the eustachian tube, and whether the video cited statistics or scientific papers. The videos were then evaluated using a five-item questionnaire adapted from the DISCERN instrument. This modified tool assessed: (1) whether the aims were clear and achieved, (2) whether the information was balanced and unbiased, (3) whether reliable sources of information were used, (4) whether additional sources of support and information were provided, and (5) whether areas of uncertainty were acknowledged. Each criterion was scored one point (range: 0-5), with higher scores reflecting greater reliability [11,12]. The five-item version follows the adaptation by Singh et al. [11] based on the original 15-item DISCERN instrument developed by Charnock et al. [12]. The original DISCERN tool demonstrated moderate inter-rater reliability, with a kappa of 0.53 (95% CI: 0.48-0.59), confirming its reproducibility among trained evaluators [12]. Data were independently collected by two medical student reviewers to ensure accuracy and consistency, with discrepancies resolved by a third reviewer.

Descriptive statistics summarized video characteristics and the frequency of specific claims and references. The prevalence of misinformation was assessed by identifying common themes and analyzing the content for unverified claims and lack of scientific validation. Additionally, the viewer engagement metrics, such as likes and comments, were analyzed to gauge the potential reach and impact of the misinformation.

In addition to video analysis, a PubMed literature search (through August 2024) used the Medical Subject Headings “otitis media” in conjunction with “Galbreath technique” (an osteopathic manipulative maneuver where mandibular movement is used to facilitate Eustachian tube drainage), “ear massaging”, “spinal adjustment”, “Eustachian tube”, and “nerve fixing”. The search included literature reviews, case reports, case series, randomized controlled trials, and basic science studies. Publications unrelated to alternative medicine in OM were excluded. A total of 14 unique publications were included in our discussion.

## Results

Video sources included chiropractors, companies, patients, and hospital systems (Table 1). Collectively, the videos accrued 2,600,209 views, had a mean duration of 191.82 seconds, and generated 21,102 likes and 1,766 comments (Table 2). Only three videos (6%) provided any scientific reference. The most frequently cited chiropractic techniques were the Galbreath technique, upper cervical spine adjustments, and ear massaging maneuvers, all of which lack robust scientific validation for OM treatment (Table 3). None of the videos mentioned evidence-based interventions such as antibiotics or tympanostomy tubes. Unverified claims regarding “fixing” nerves were present in 14 videos (28%), whereas “fixing” the Eustachian tube was asserted in 25 videos (50%). Despite the prevalence of misinformation, viewer engagement was substantial, with an average of 431 likes and 35 comments per video. The total mean modified DISCERN score was 1.0 ± 0.9 out of 5. Companies/advertisements had the highest mean modified DISCERN score, followed by hospital systems. Chiropractors and patient testimonials received the lowest mean scores (Table 4).

**Table 1:** Video Creator Type

Video Creator	Videos Analyzed, N (%)
Chiropractor	42 (84.0)
Company/Advertisement	4 (8.0)
Patient Testimonial	2 (4.0)
Hospital	2 (4.0)
Total	50 (100.0)

**Table 2:** Viewer-Engagement Metrics for YouTube Videos by Creator Type Total numbers of views, likes, and comments accrued by each creator category, demonstrating that chiropractor-produced videos accounted for the vast majority of engagement.

Video Creator	Number of Views	Likes	Comments
Chiropractor	2,523,745	20,755	1,756
Company/Advertiser	21,173	182	9
Patient Testimonial	40,223	110	1
Hospital	15,102	55	0
Total	2,600,209	21,102	1,766

**Table 3:** Frequency of Chiropractic and Osteopathic Techniques Mentioned in YouTube Videos and the Corresponding Published Literature *The literature counts derive from the PubMed search described in the Methods section. Numbers represent how many peer-reviewed publications discuss or evaluate each technique. The specific studies corresponding to these counts are cited and discussed in the Discussion section. Listing a study does not imply clinical efficacy. Number and percentage of videos referencing each technique alongside the count of peer-reviewed publications discussing that technique, highlighting the discrepancy between online promotion and available evidence.

Technique Mentioned in YouTube Videos	Videos, N (%)	Published Literature*
Upper Cervical Spine Adjustments	32 (64.0)	10 studies
Ear Massaging	25 (50.0)	1 study
Lymphatic Massage	1 (2.0)	13 studies
Galbreath Technique	1 (2.0)	12 studies

**Table 4:** Video Sources and Mean Modified DISCERN Score

Source	N (%)	Modified DISCERN Score ± SD
Chiropractor	42 (84.0)	0.9 ± 0.8
Company/advertisement	4 (8.0)	2.0 ± 1.2
Patient testimonial	2 (4.0)	0.8 ± 0.3
Hospital	2 (4.0)	1.5 ± 0.5

In this sample, 42 videos (84%) originated from chiropractic or commercial channels, whereas only two videos (4%) came from hospital or academic sources (Table 1).

## Discussion

Scientific evidence gaps and associated risks

The chiropractic techniques highlighted, upper cervical spine adjustments, the Galbreath maneuver, and peri-auricular massage, have limited clinical support. A 2013 systematic review of osteopathic and chiropractic interventions for pediatric conditions found insufficient or low-quality data to support manipulative therapy for OM [3]. A broader 2016 review reached the same conclusion, identifying no randomized controlled trial (RCT) demonstrating meaningful clinical benefit [4]. The Galbreath maneuver is supported by a single case report in which a 14-month-old received both the technique and a 10-day course of amoxicillin, precluding causal inference [13]. No clinical trials have evaluated ear massage. Spinal manipulative therapy has not shown efficacy for OM and carries a documented, though rare, risk of vertebral artery dissection and stroke [14]. Untreated or undertreated OM increases the risk of chronic effusion, conductive hearing loss, and speech-language delay [15]; thus, misinformation that discourages proven treatments poses tangible clinical risks.

Existing clinical evidence 

Several manual techniques highlighted in YouTube videos have also been described in the scientific literature. Lymphatic massage has appeared in 13 studies [3,4,13-23], while the Galbreath technique was reported in 12 studies [3,4,13-18,20-23]. Upper cervical spine adjustments have been discussed or evaluated in 10 studies [3,4,13-17,21-23]. Ear massaging was identified in one study [15]. Although these techniques are represented in the literature, the number of peer-reviewed evaluations is modest compared with the frequency of online promotion, and published studies vary widely in quality and clinical relevance.

Our PubMed search identified 14 publications on chiropractic and osteopathic techniques for OM. Most were small studies, including case reports and limited randomized trials, with methodological weaknesses such as small sample size, short follow-up, and lack of controls [3,4,16]. A few pilot trials suggested modest benefit, but overall evidence remains inconclusive. This limited literature contrasts with the confident claims promoted on YouTube.

Existing clinical evidence systematic reviews consistently conclude that evidence supporting chiropractic or osteopathic manipulation for OM is weak. A recent scoping review identified three RCTs and one small cohort study (total N≈205 pediatric patients aged approximately six months to six years) examining osteopathic manipulative treatment (OMT) for OM [5]. This review concluded, with low certainty, that OMT may modestly reduce recurrence rates and improve middle ear function; however, the evidence remains limited due to small sample sizes and methodological weaknesses.

Mills et al. [17] conducted an RCT involving 57 children comparing OMT plus standard care versus standard care alone. They found that the OMT group experienced fewer OM episodes (-0.14 episodes/month; p=0.04), fewer surgeries (1 vs. 8 surgeries; p=0.03), and more surgery-free months (6.0 vs. 5.25 months; p=0.01). However, this study was limited by its small sample size, restricting generalizability and definitive conclusions [17]. Steele et al. [18] performed a pilot RCT with 52 children (43 completed), providing three weekly OMT sessions in addition to standard care. They observed faster resolution of middle-ear effusion at three weeks (OR 2.98; 95% CI: 1.16-7.62; p=0.02) [18]. Degenhardt et al. conducted a small cohort study with eight pediatric patients, noting 62.5% avoided OM recurrence at one year. However, the lack of a control group precluded any causal inference [19].

The chiropractic literature regarding OM is even more limited. A systematic review identified only one weak RCT, two observational studies, 36 case reports, and 17 conference abstracts related to spinal manipulation in pediatric OM, with substantial methodological weaknesses such as small sample sizes, lack of blinding, absence of adequate controls, and short follow-up durations [16].

Common limitations across these studies included small sample sizes, short follow-up periods, methodological weaknesses (e.g., lack of blinding and lack of control group), substantial heterogeneity in OMT protocols, and a lack of reporting on adverse events. These gaps in the literature, including limited trials, significant methodological flaws, and heterogeneity, illustrate why claims promoting chiropractic or osteopathic manipulation for OM in YouTube videos are unsupported by robust clinical data.

Public health implications

Widely viewed misinformation can erode trust in clinicians and reduce adherence to evidence-based care [20]. Caregivers may instead pursue unproven chiropractic treatments, delaying effective therapy and incurring unnecessary costs. Prior reviews highlight ethical concerns when commercial promotion precedes conclusive evidence [16]. The Galbreath maneuver, often cited online, is supported by only a single case report showing short-term improvement, likely reflecting the natural resolution of acute OM [13]. The overall paucity of high-level evidence for chiropractic interventions in OM highlights the danger of promoting such therapies online.

Role of digital platforms

YouTube’s recommendation algorithm prioritizes engagement, often boosting sensational claims [21,22]. As found here, 84% of videos came from commercial or chiropractic channels, and only 4% from academic or hospital sources (Table 1). Studies of other medical topics show similar trends, with credible content frequently buried beneath anecdotal or advertisement-driven videos [23]. Popular health-related videos can match expert-led content in understandability and presentation quality, making it difficult for viewers to discern that they often contain misinformation and commercial bias [23]. Greater collaboration between platform moderators and medical organizations is needed to boost the visibility of reliable, evidence-based content.

Limitations

A key limitation of this study is the reliance on a single time-point search (January 2023), which may not fully capture changes in YouTube’s search algorithms or the dynamic nature of video availability. As such, the reproducibility of our findings over time may be limited. In addition, while our analysis highlights a problem that is widely suspected, the prevalence of inaccurate medical information on social media, our contribution lies in measuring prevalence, mapping recurring themes, and comparing them against established scientific evidence. By documenting these discrepancies, we provide concrete data that can inform media literacy efforts and policy interventions.

Future directions for research and policy

RCTs pairing manipulative therapy with standard care could clarify whether any complementary benefit exists, but existing pilot studies remain limited by small sample sizes, high attrition, and heterogeneous protocols [3,4]. Future implementation studies should pilot brief media literacy workshops teaching source evaluation alongside real-time fact check banners on video platforms and then measure changes in users’ ability to identify and resist misinformation via assessments. The American Academy of Otolaryngology - Head and Neck Surgery and allied organizations could develop search engine-optimized videos addressing common OM questions to counterbalance misleading content. Finally, clearer platform policies that demote or label content contradicting established clinical guidelines may help protect the public while preserving open discourse.

## Conclusions

This cross-sectional analysis reveals that YouTube contains a high concentration of misinformation regarding chiropractic management of OM. These videos attract significant public engagement despite omitting evidence-based therapies, lacking scientific citations, and promoting unproven claims such as “fixing” nerves or the Eustachian tube. Because untreated OM can lead to hearing loss and developmental delays, the propagation of such misinformation poses measurable health risks. Health professionals, medical societies, and digital platforms must collaborate to increase access to reliable content, implement media literacy strategies, and develop policies that demote misleading medical videos. Further randomized trials are necessary to evaluate whether chiropractic or osteopathic techniques provide any benefit when combined with standard care. Until such data exist, clinicians should counsel caregivers that spinal or cranial manipulation remains unproven for OM. A coordinated, multi-stakeholder response is essential to protect children and their caregivers from the harms of online health misinformation.
